# Distribution of Preterm Birth History Among Puerto Rican Children With Asthma: A Rural-Urban Comparison in a Pediatric Pulmonology Cohort

**DOI:** 10.7759/cureus.112923

**Published:** 2026-07-18

**Authors:** Bryan J Calderin Barreto, Virna M Nazario Ayala, Leyra L Vazquez, Ivan R Iriarte, Martha L Villarreal Morales, Marina Martinez Garri, Carolina Miranda Torres, Liza C Sánchez Plazas

**Affiliations:** 1 Pediatrics, Puerto Rico Children's Hospital, Bayamón, PRI; 2 Pediatrics and Neonatology, Ponce Health Sciences University, Ponce, PRI; 3 Family Medicine, Mayagüez Medical Center, Mayagüez, PRI; 4 Research, Puerto Rico Children's Hospital, Bayamón, PRI; 5 Pulmonology, Puerto Rico Children's Hospital, Bayamón, PRI; 6 Pediatrics and Neonatology, Puerto Rico Children's Hospital, Bayamón, PRI

**Keywords:** childhood asthma, gestational age, pediatric asthma, prematurity, preterm birth, puerto rico, rural health, urban health

## Abstract

Preterm birth (<37 weeks of gestation) has been associated with altered lung development and increased risk of childhood asthma. Puerto Rico has a high burden of both prematurity and childhood asthma; however, the distribution of prematurity among children with asthma across geographic regions remains insufficiently characterized. We conducted a retrospective cross-sectional study of children aged 2-11 years with a confirmed asthma diagnosis followed at a pediatric pulmonology clinic in Bayamón, Puerto Rico. Demographic, clinical, environmental, and gestational data were extracted from electronic medical records. The proportion of preterm births was calculated with 95% CIs, and rural-urban differences were assessed using a chi-square test and ORs. Among 294 eligible participants, gestational age data were available for 281 children. Forty-one children (14.6%; 95% CI: 10.5%-19.8%) were born preterm. Prematurity occurred in 16.3% of rural participants and 12.4% of urban participants, with no statistically significant difference detected (χ² = 0.54, p = 0.46; OR = 0.73, 95% CI: 0.37-1.45). These findings describe the distribution of preterm birth history among children with asthma followed at a pediatric pulmonology clinic and do not demonstrate a statistically significant rural-urban difference, though the estimate was imprecise and a clinically meaningful difference cannot be excluded.

## Introduction

Preterm birth (<37 weeks of gestation) is a major global health burden and remains an important contributor to neonatal and under-five mortality, while also being associated with long-term morbidity across the life course [1]. Preterm birth has been linked to impaired lung development, reduced lung function, and increased risk of respiratory conditions during childhood and adolescence, including recurrent wheeze and asthma [2,3]. Large population-based studies demonstrate that individuals born preterm have an increased risk of asthma from childhood into adulthood [4], and nationwide register data further support increased respiratory risk extending into adult life [5].

Asthma is one of the most common chronic diseases in childhood and represents a major global public health problem [6]. Puerto Rican children experience a disproportionate asthma burden compared with other groups in the United States [7,8], and prior research suggests that place of residence may influence asthma outcomes in this population [7]. Puerto Rico also has persistently elevated prematurity rates. The 2025 March of Dimes Puerto Rico report card documented a preterm birth rate of 12.2% for 2024 births [9].

Prior island-based work has examined the relationship between prematurity and asthma in Puerto Rican children, with atopy identified as a potential modifying factor [10]. Environmental exposures remain highly prevalent in Puerto Rico and may contribute to asthma morbidity [11]. Rural and urban environments may differ in environmental exposures and social determinants of health, which could influence asthma phenotypes and disease burden. Therefore, this study aimed to describe the prevalence of preterm birth among Puerto Rican children with a confirmed asthma diagnosis followed at a pediatric pulmonology clinic and to evaluate whether the distribution of prematurity differs between rural and urban regions within this clinical cohort.

## Materials and methods

A retrospective cross-sectional study was conducted by reviewing electronic medical records (EMRs) between April 2025 and July 2025. The study included male and female children aged 2-11 years with a confirmed asthma diagnosis who were followed at a pediatric pulmonology clinic in Bayamón, Puerto Rico. The clinic provides care to patients residing in Bayamón and surrounding municipalities, including Guaynabo, Toa Baja, Toa Alta, Naranjito, Corozal, Comerío, Cataño, San Juan, and neighboring areas. Patients with concomitant chronic pulmonary conditions or diagnoses likely to confound respiratory outcomes were excluded, including cystic fibrosis, neuromuscular disorders, primary ciliary dyskinesia, ventilator dependence, interstitial lung disease, congenital pulmonary malformations, and anatomical airway anomalies.

Eligible cases were identified using International Classification of Diseases, Tenth Revision, Clinical Modification (ICD-10-CM) asthma diagnostic codes, including unspecified asthma (J45.9), mild intermittent asthma (J45.20), mild persistent asthma (J45.30), moderate intermittent asthma (J45.22), moderate persistent asthma (J45.40), severe asthma (J45.5), severe persistent asthma (J45.50), and other asthma (J45.998), an approach consistent with validated diagnostic coding algorithms for identifying childhood asthma in administrative records [12]. For children aged 6 years and older, an asthma diagnosis was supported by spirometric evidence of reversible airflow obstruction where available. For children aged 2-5 years, in whom spirometry is not reliably performed, asthma diagnosis was based on physician clinical assessment documented in the EMR, consistent with accepted diagnostic approaches for preschool-aged children [13]. These criteria included recurrent episodes of wheezing, cough, or breathing difficulty, response to bronchodilator therapy, and exclusion of alternative diagnoses, as assessed by the treating pediatric pulmonologist at the time of the clinic visit. Data were extracted using a standardized data collection form. Variables collected included sex, age at evaluation, age at asthma diagnosis, family history of asthma, gestational age at birth, environmental exposures (including pet or animal exposure, tobacco smoke or vaping exposure, and dust or carpet exposure), and municipality of residence.

Geographic classification (rural vs urban) was determined based on the municipality of residence as documented in the EMR at the time of patient registration, following local administrative designations. Municipal classification followed local administrative designations rather than census-tract-level analysis. Urban municipalities included Bayamón, San Juan, Guaynabo, Toa Baja, Carolina, Caguas, Trujillo Alto, and Ponce; all remaining municipalities were classified as rural. It should be noted that the current municipality of residence may not reflect the municipality of residence at birth, which represents a potential limitation of the geographic classification used in this study.

The primary outcome was the documented history of preterm birth, defined as gestational age less than 37 completed weeks. Gestational age was further categorized into extreme preterm (<28 weeks), very preterm (28-31 weeks), early preterm (32-33 weeks), late preterm (34-36 weeks), and term (≥37 weeks). Continuous variables were summarized using mean, SD, median, and range after inspection for distribution symmetry, and categorical variables were presented as frequencies and percentages.

The proportion of children with a documented history of preterm birth was calculated, along with corresponding 95% CIs, using the exact binomial method. Of the 294 eligible participants, gestational age data were available for 281 children; 13 participants with missing gestational age data were excluded from prematurity-specific analyses but retained for other descriptive analyses. Missing data across other variables reflected documentation gaps in the EMR: age at evaluation was unavailable for 11 participants due to non-standard date-of-birth formatting identified during data cleaning; age at asthma diagnosis was missing for 15 participants who lacked a documented diagnosis date; municipality of residence was missing for 15 participants whose registration records lacked a documented place of residence; and family history of asthma was missing for two participants whose charts contained no documentation of this variable. Environmental exposure data had near-complete ascertainment (N = 293) because the absence of a documented exposure was coded as no exposure per the standardized data extraction protocol.

To evaluate differences in the distribution of prematurity between rural and urban regions, a chi-square test of independence was performed. When expected cell counts were less than five, Fisher’s exact test was applied. ORs with 95% CIs were calculated to estimate the strength of association between geographic setting and prematurity. The absolute prevalence difference between rural and urban groups was calculated with its 95% CI using the Wilson score method for the difference between two proportions. All statistical tests were two-sided, and a p-value <0.05 was considered statistically significant. Statistical analyses were performed using MedCalc^®^ version 23.2.7 (MedCalc Software Ltd, Ostend, Belgium). This study was conducted and reported in accordance with the Strengthening the Reporting of Observational Studies in Epidemiology (STROBE) guidelines [14].

All data were de-identified at the time of extraction and stored in a secure database accessible only to authorized study personnel in accordance with institutional privacy and ethical guidelines. The study was reviewed and approved by the Ponce Research Institute Institutional Review Board (protocol #2504259746). Informed consent was waived, given the retrospective, de-identified design.

## Results

A total of 294 children aged 2-11 years with a confirmed asthma diagnosis were included in the analysis. Baseline demographic and clinical characteristics stratified by geographic setting are summarized in Table 1.

**Table 1:** Baseline demographic and clinical characteristics of the study population stratified by geographic setting (N = 293). Values are presented as n (%) unless otherwise indicated. Continuous variables are expressed as mean ± SD and median (range). One participant lacked geographic classification data and is excluded from this table; total cohort N = 294. Age data were available for 164 rural and 119 urban participants; age at diagnosis data for 161 rural and 118 urban participants; and family history data for 167 rural and 125 urban participants. Geographic classification was based on the documented municipality of residence.

Variable	Rural (N = 168)	Urban (N = 125)	Total (N = 293)
Gender			
Male	95 (56.5%)	75 (60.0%)	170 (58.0%)
Female	73 (43.5%)	50 (40.0%)	123 (42.0%)
Age at evaluation (years)			
Mean ± SD	5.5 ± 2.3	5.7 ± 2.3	5.6 ± 2.3
Median (range)	5 (2-11)	5 (2-11)	5 (2-11)
2-5 years	83 (50.6%)	61 (51.3%)	144 (50.9%)
6-11 years	81 (49.4%)	58 (48.7%)	139 (49.1%)
Age at asthma diagnosis (years)			
Mean ± SD	4.3 ± 2.3	3.7 ± 2.1	4.1 ± 2.3
Median (range)	4 (0.42-10)	4 (0.42-10)	4 (0.42-10)
Family history of asthma			
Yes	93 (55.7%)	75 (60.0%)	168 (57.5%)
No	74 (44.3%)	50 (40.0%)	124 (42.5%)
Environmental exposures			
Pets or animals	72 (42.9%)	55 (44.0%)	127 (43.3%)
Tobacco smoke or vaping	19 (11.3%)	13 (10.4%)	32 (10.9%)
Dust or carpet	4 (2.4%)	5 (4.0%)	9 (3.1%)
Gestational age available	160 (95.2%)	121 (96.8%)	281 (95.9%)
Preterm birth	26 (16.3%)	15 (12.4%)	41 (14.6%)

**Figure 1:**
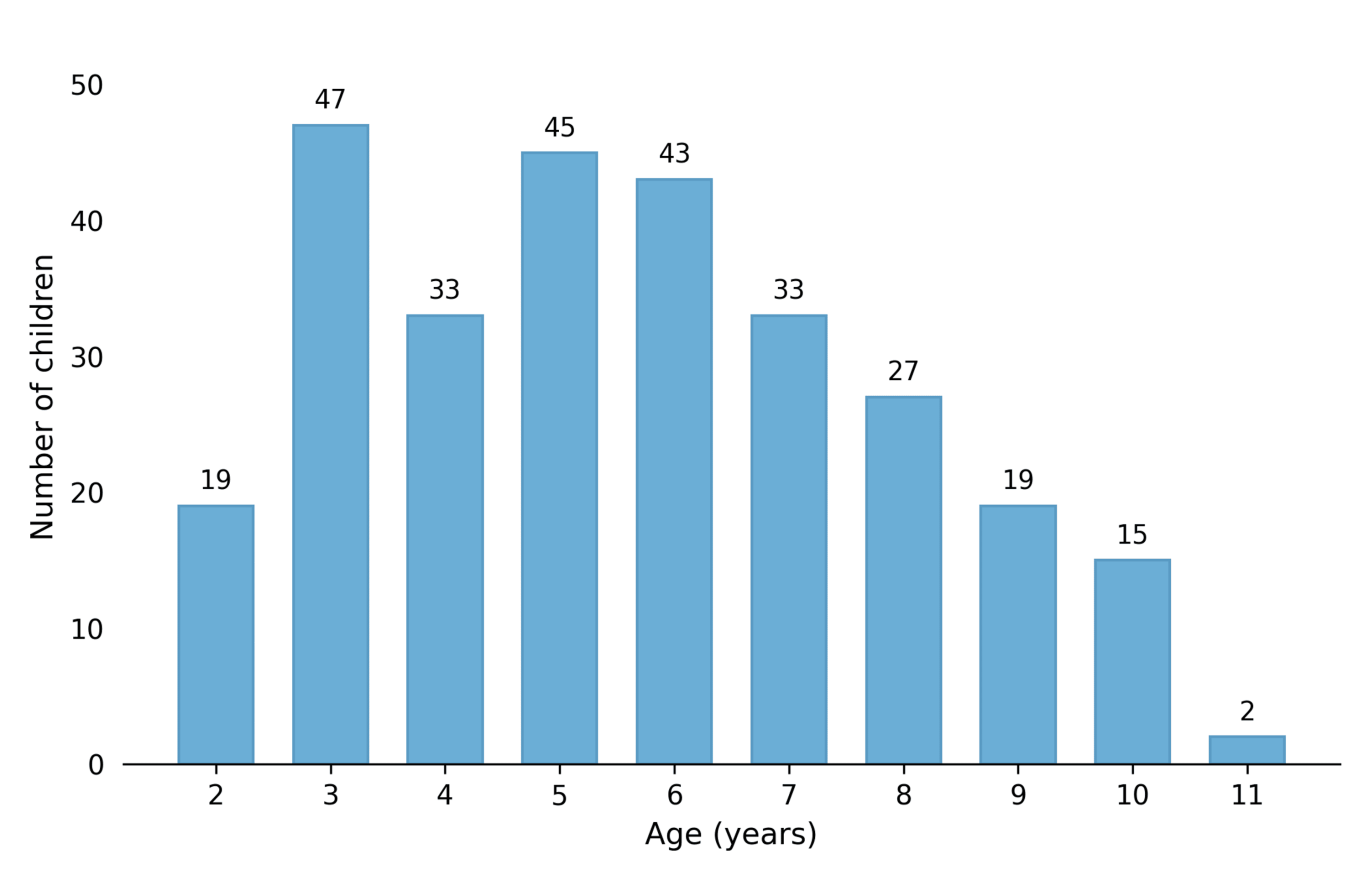
Frequency distribution of age at evaluation among children with asthma (N = 283). Bar chart showing the frequency distribution of age at evaluation among 283 children with asthma. Age is displayed on the horizontal axis in years, and the number of children is shown on the vertical axis.

The cohort was predominantly male, with 170 (58.0%) males and 123 (42.0%) females. Age data were available for 283 participants due to formatting inconsistencies identified during data cleaning. The mean age at evaluation was 5.6 years (median 5 years, SD 2.3; range 2-11 years). When grouped, 144 of 283 children (50.9%) were between 2 and 5 years old, and 139 of 283 (49.1%) were between 6 and 11 years old. The age distribution is illustrated in Figure 1.

**Figure 2:**
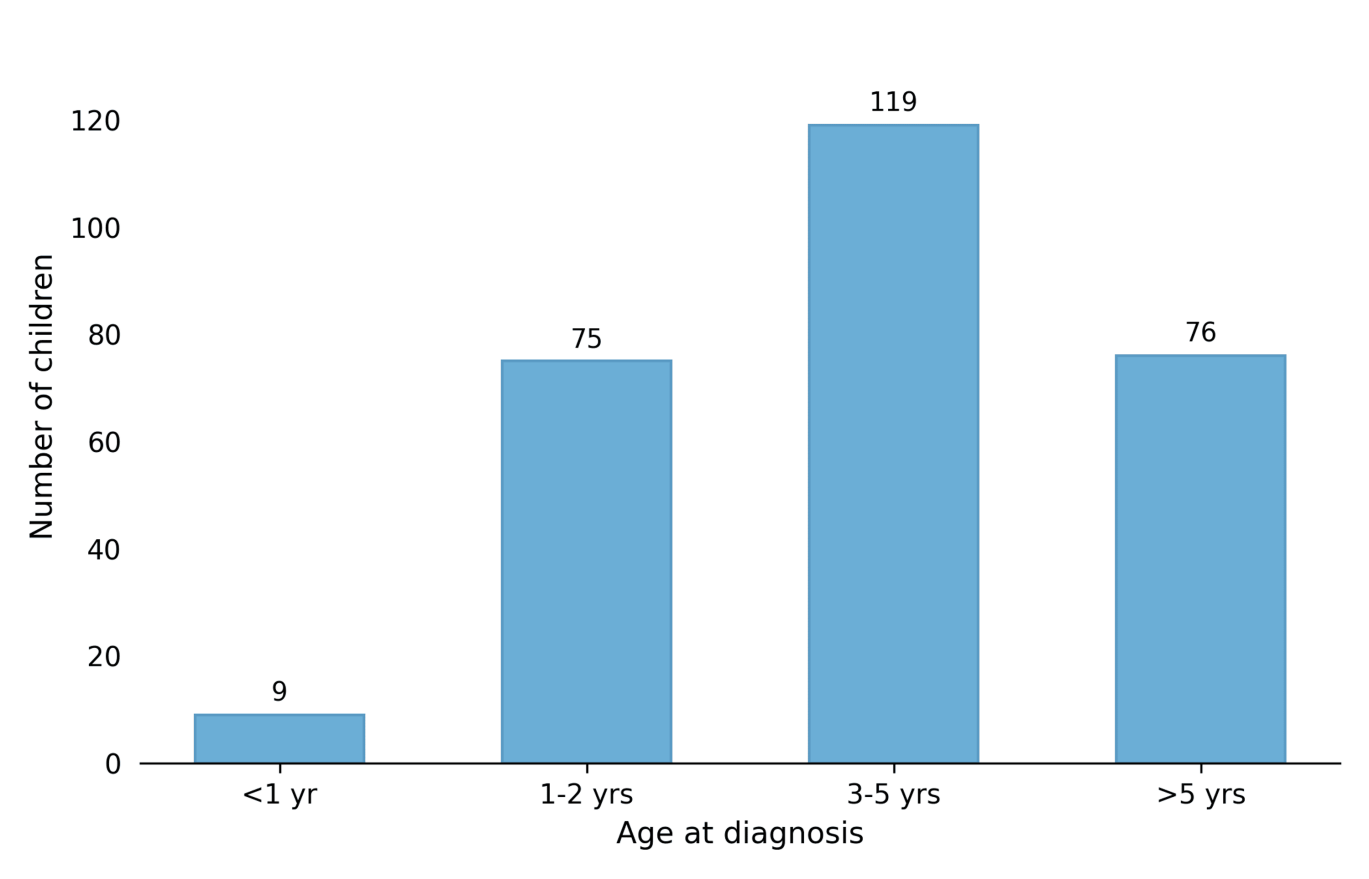
Distribution of age at asthma diagnosis (N = 279). Bar chart showing age categories at asthma diagnosis among 279 participants. Age groups are displayed on the horizontal axis, and the number of children is shown on the vertical axis. The category “>5 years” includes children diagnosed between the ages of 6 and 10.

Age at asthma diagnosis data were available for 279 participants. The mean age at diagnosis was 4.1 years (median 4 years, SD 2.3; range 0.42-10 years). The largest group (119/279, 42.7%) received a diagnosis between ages 3 and 5. Additionally, 75 children (26.9%) were diagnosed between 1 and 2 years of age, and nine children (3.2%) were diagnosed within the first year of life. The distribution of age at diagnosis is presented in Figure 2.

A positive family history of asthma was documented in 168 of 292 participants (57.5%). With respect to geographic classification, 168 of 294 children (57.1%) resided in rural regions and 125 (42.7%) in urban settings, as shown in Table 1.

Environmental exposures were common within the cohort. Exposure to pets or animals was reported in 127 of 294 participants (43.2%), tobacco smoke or vaping exposure in 32 (10.9%), and dust or carpet exposure in nine (3.1%). Nearly half of the participants (142/294, 48.3%) had no documented environmental exposures. Seventeen children (5.8%) were exposed to two or more exposure categories simultaneously. These exposure data are summarized in Table 1.

**Figure 3:**
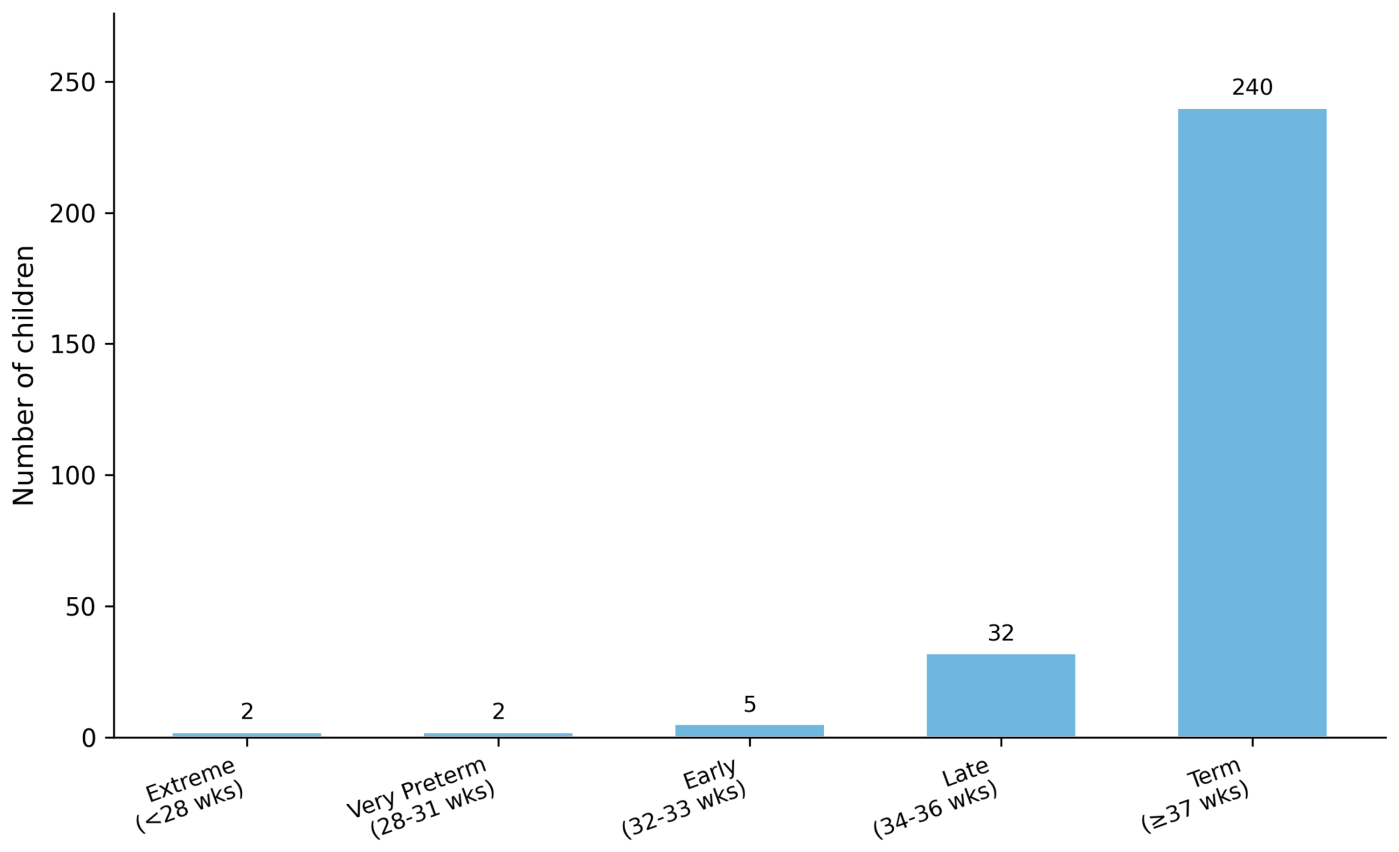
Gestational age distribution among participants with available data (N = 281). Bar chart illustrating gestational age categories among 281 children with available gestational age data. Preterm birth was defined as <37 completed weeks of gestation.

Gestational age data were available for 281 participants. Among these, 41 children (14.6%; 95% CI: 10.5%-19.8%) had a documented history of preterm birth (<37 weeks), while 240 (85.4%) were born at term. Within the preterm group, two children (0.7%) were classified as extreme preterm (<28 weeks), two (0.7%) as very preterm (28-31 weeks), five (1.8%) as early preterm (32-33 weeks), and 32 (11.4%) as late preterm (34-36 weeks). The distribution of gestational age categories is shown in Figure 3, and a detailed breakdown by gestational age is provided in Table 2.

**Table 2:** Gestational age distribution among participants with available data (N = 281). Preterm birth was defined as <37 completed weeks of gestation. Analysis includes 281 participants with available gestational age data; 13 participants were excluded due to missing gestational age documentation.

Gestational category	N (%)
Extreme preterm (<28 weeks)	2 (0.7%)
Very preterm (28-31 weeks)	2 (0.7%)
Early preterm (32-33 weeks)	5 (1.8%)
Late preterm (34-36 weeks)	32 (11.4%)
Term (≥37 weeks)	240 (85.4%)
Total preterm (<37 weeks)	41 (14.6%)

When stratified by geographic setting among participants with available gestational age data (N = 281), 26 of 160 children in rural regions (16.3%) had a documented history of preterm birth compared with 15 of 121 children in urban regions (12.4%). The absolute prevalence difference was 3.9 percentage points (95% CI: -4.0% to 11.8%), indicating that the estimate was imprecise and a clinically meaningful difference cannot be excluded. This difference was not statistically significant (χ² = 0.54, p = 0.46). The calculated OR for a documented history of preterm birth in urban versus rural regions was 0.73 (95% CI: 0.37-1.45). These findings are summarized in Table 3.

**Table 3:** Distribution of documented preterm birth history by geographic setting (N = 281). Preterm birth was defined as <37 completed weeks of gestation. Analysis restricted to participants with available gestational age data (N = 281). Chi-square test of independence was performed (χ² = 0.54, p = 0.46). OR (urban vs rural) = 0.73 (95% CI: 0.37-1.45). Absolute prevalence difference (rural minus urban) = 3.9 percentage points (95% CI: -4.0% to 11.8%).

Geographic setting	Preterm N (%)	Term N (%)	Total
Rural	26 (16.3%)	134 (83.7%)	160
Urban	15 (12.4%)	106 (87.6%)	121

**Figure 4:**
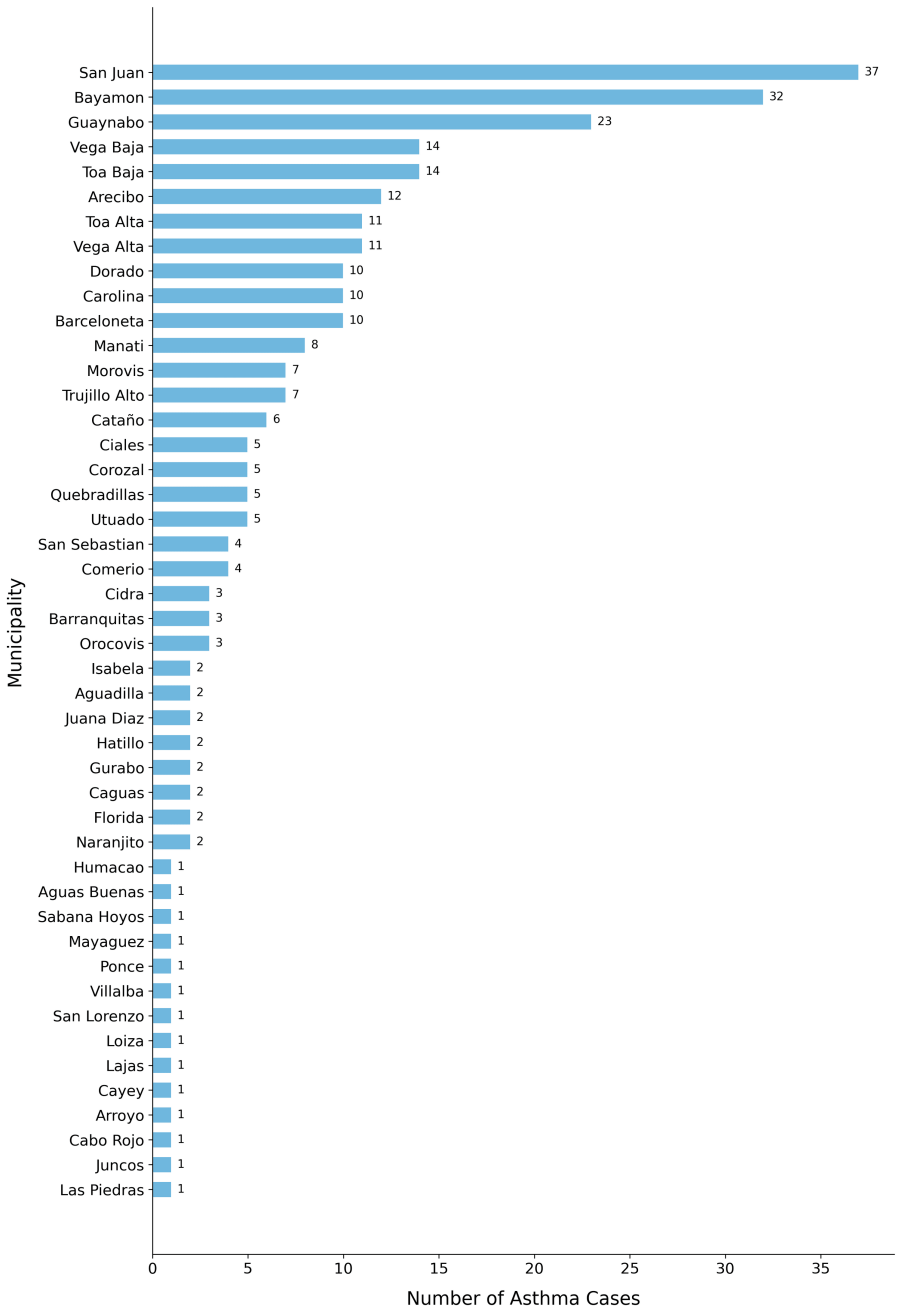
Distribution of asthma cases by municipality of residence (absolute counts). Bar chart illustrating the number of children diagnosed with asthma included in this study by municipality of residence. Municipalities are displayed on the vertical axis, and absolute case counts are shown on the horizontal axis. Data reflect 279 participants with available municipality information; 15 records lacked documented municipality data. Case counts reflect clinic referral patterns and do not represent population-level estimates of asthma burden.

Municipality of residence was available for 279 of 294 participants (94.9%), with 15 records lacking documented municipality information. The distribution of cases by municipality is illustrated in Figure 4. Absolute case counts by municipality reflect clinic referral patterns and should be interpreted in the context of the catchment area served by this pediatric pulmonology clinic, rather than as population-level estimates of asthma burden.

## Discussion

This study examined the documented history of preterm birth among Puerto Rican children with asthma followed at a pediatric pulmonology clinic and evaluated whether the distribution of prematurity differed by geographic setting. Within this clinical cohort, 14.6% (95% CI: 10.5%-19.8%) of children with available gestational age data had a documented history of preterm birth. This estimate falls within the range of contemporary population-level prematurity rates reported for Puerto Rico, including the 12.2% rate documented in the 2025 March of Dimes Puerto Rico report card [9]. The overlap between these values limits the ability to infer that prematurity is disproportionately represented among children with asthma in this clinical sample, particularly in the absence of a non-asthmatic comparison group.

Preterm birth has been consistently associated with increased risk of wheezing disorders and physician-diagnosed asthma in childhood. A large meta-analysis demonstrated that children born preterm have significantly higher odds of developing wheezing disorders and asthma compared with term counterparts, with greater risk observed at lower gestational ages [3]. Large population-based cohort studies further support that this increased respiratory risk extends into adolescence and adulthood [4], and nationwide register data confirm persistent associations between preterm birth and chronic respiratory conditions into adult life [5]. These findings reinforce the biological plausibility of the link between prematurity and long-term airway vulnerability. However, the present study was not designed to evaluate this association, as it includes only children with a confirmed diagnosis of asthma and lacks a non-asthmatic comparison group.

Despite this established association in the broader literature, the predominance of late preterm births (34-36 weeks) in our cohort is noteworthy. Late preterm infants typically experience less severe pulmonary immaturity compared with very or extremely preterm infants, potentially resulting in a more modest contribution to respiratory morbidity relative to earlier gestational categories [15]. This distribution may partially explain the similarity between the observed prevalence of prematurity in this cohort and population-level estimates for Puerto Rico.

When stratified by geographic setting, a documented history of preterm birth was present in 16.3% of children in rural regions and 12.4% in urban regions. Although numerically higher among rural participants, the absolute prevalence difference was 3.9 percentage points (95% CI: -4.0% to 11.8%) and was not statistically significant (χ² = 0.54, p = 0.46). The wide CI around both the prevalence difference and the OR (OR = 0.73; 95% CI: 0.37-1.45) indicates that the study did not estimate the difference with sufficient precision to exclude a clinically meaningful rural-urban disparity. These findings should therefore not be interpreted as evidence that no geographic difference exists, but rather that this study was underpowered to detect one if present. Prior research has described geographic variation in asthma burden among Puerto Rican children [7]; however, the present study was not designed to evaluate whether prematurity explains this variation, as it includes only children with asthma and lacks population-level denominators.

Asthma in Puerto Rican children is widely recognized as multifactorial. Puerto Rican children bear a disproportionate asthma burden compared with other racial and ethnic groups in the United States [7,8], a disparity that cannot be attributed to a single perinatal factor. In this study, 57.5% of participants had a documented family history of asthma, underscoring the contribution of genetic susceptibility, consistent with genome-wide association data identifying asthma-associated loci in Latino populations [16]. Environmental exposures were also prevalent, including pet or animal exposure and tobacco smoke or vaping exposure. Prior Puerto Rico-based research has documented the frequency of household environmental exposures and their association with asthma morbidity [11]. These factors likely interact with perinatal determinants and social determinants of health in ways that the present study was not designed to disentangle.

The findings of this study should be interpreted in the context of several methodological considerations. In this retrospective, single-center chart review, analyses relied on the accuracy and completeness of EMR documentation. Gestational age data were unavailable for 13 participants, resulting in exclusion from prematurity-specific analyses. Additionally, the municipality of residence was unavailable for 15 participants, which may slightly limit the cohort’s geographic representation. Importantly, the current municipality of residence may not reflect the municipality of residence at birth, which limits the precision of the geographic classification used in this study. The absence of a non-asthmatic control group precludes direct comparison of prematurity prevalence between children with and without asthma and limits causal inference. Pulmonary function testing was not uniformly available in younger children, which may introduce diagnostic variability, particularly among preschool-aged participants in whom asthma diagnosis may overlap with transient wheezing phenotypes [17]. Geographic classification was based on municipal designation and broadly categorized as rural or urban; this binary classification may not fully capture within-region heterogeneity in socioeconomic conditions, environmental exposures, or access to healthcare services. Although rural and urban groups were compared statistically, the relatively small sample size and limited number of preterm cases (N = 41) may have restricted statistical power to detect modest geographic differences, as reflected in the wide CIs observed. Furthermore, because participants were recruited from a specialty pediatric pulmonology clinic, the cohort may overrepresent children with more persistent or complex asthma, potentially limiting its generalizability to the broader pediatric population in Puerto Rico. Additionally, the island has a limited number of pediatric pulmonologists, and access to subspecialty care varies considerably with insurance coverage, which may disproportionately affect children from lower-income households [18]. As a result, a subset of children with asthma, including those born prematurely, may not have had the opportunity to reach specialty evaluation and receive a formal diagnosis, potentially leading to under-ascertainment of asthma cases in this population.

Globally, asthma remains a significant public health condition with persistent morbidity across pediatric populations [6]. The findings of the present study contribute descriptive data on the prevalence and geographic distribution of documented preterm birth history within a clinic-based cohort of Puerto Rican children with asthma. Future population-based investigations incorporating non-asthmatic comparison groups, population-level denominators, and multivariable modeling are warranted to clarify the independent contribution of prematurity to asthma risk and geographic variation in asthma burden among Puerto Rican children.

## Conclusions

In this retrospective cross-sectional study of Puerto Rican children with a confirmed asthma diagnosis followed at a pediatric pulmonology clinic in Bayamón, Puerto Rico, 14.6% of participants with available gestational age data had a documented history of preterm birth. This proportion is consistent with reported population-level prematurity rates for Puerto Rico. The distribution of documented preterm birth history did not differ significantly between rural and urban regions within this clinical cohort; however, the wide CIs observed indicate that the study was not sufficiently powered to exclude a clinically meaningful geographic difference. These findings are descriptive and specific to this clinic-based cohort; they cannot determine whether prematurity contributes to asthma risk or explains geographic variation in asthma burden, as no non-asthmatic comparison group was included. Future prospective, population-based investigations incorporating non-asthmatic comparison groups, population-level denominators, and multivariable analyses are needed to clarify the relationship between prematurity, geographic setting, and asthma risk in Puerto Rican children.
